# On Medical Reporting

**Published:** 1829-07-01

**Authors:** 


### XXVII.

### MEDICAL REPORTING.

In our last number, page 513, we made some observations on the evil consequences attendant on the present, or rather we hope the late system of medical reporting. In the London Medical Society, Dr. Clutterbuck brought forward a motion, at a special general meeting, for the entire suppression of medical reports of their proceedings. Dr. C. very ably exposed the many bad consequences to which the system of reporting led, and some of which we had previously pointed out. Had the worthy Doctor limited his motion to a correction of the evil rather than to a total suppression of reports, he would have had nine out of ten votes in his favour. Even as it was the votes were even, and the president gave his *additional,* or casting vote, against Dr. Clutterbuck.

The society then left it to the council to draw up a plan for the regulation of reporting, and the following appears to be the substance of the regulations agreed upon, and read to the Society.

1*mo*. The reporter must be a medical man, and he is not to take any part in the debates of the society.

2*ndo*. A reporter from each journal is to be admitted only by a card from the president, which admission is to continue *during the pleasure of the president*—in other words, as long as the reporter "se bene gesserit," that is, reports the truth, and nothing but the truth.

3*tio*. If any gentleman in the society, when stating a case, requests that his own name, or that of the patient, may be kept back in the report, his request is to be complied with by the reporter.

By the foregoing regulation there is full permission given to journals to report "the truth, the whole truth, and nothing but the truth." We think that such permission ought to satisfy the most strenuous advocate for the liberty of the press. We grant, indeed, that such a regulation must prove a very great annoyance to some journals, where the garnish of scandal, falsehood, and misrepresentation, form the grand attraction to the literary banquet. How will the profession stare to see the blushing Goddess of TRUTH figuring in the pages of the Lancet! The phenomenon will not be less surprising than the presence of Catholics in Parliament! The goddess, to be sure, will find herself occasionally in suspicious company; but who knows what wonders she may work by the mere force of example?

We hope the WESTMINSTER Medical Society will follow the salutary example set them by the LONDON.

---

